# Conceptualisation of African primal health care within mental health care

**DOI:** 10.4102/curationis.v41i1.1753

**Published:** 2018-03-22

**Authors:** Neo E. Nare, Abel J. Pienaar, Ditaba D. Mphuthi

**Affiliations:** 1 School of Nursing Science, North-West University, South Africa; 2 Department of Health Studies, University of South Africa, South Africa

## Abstract

**Background:**

It is believed by western education systems that the first contact should be with the nurse in primary health care. However, it is not the case. Therefore, the researcher attempts to correct this misconception by conceptualising the correct beginning of health seeking behaviour in an indigenous African community, namely African Primal Health Care (APHC). ‘Primal’ was coined during a colloquium by Dr Mbulawa and Seboka team members; however no formal conceptualisation took place, only operational definition. Due to the study scope, conceptualisation is narrowed to mental health, but this concept is applicable in the broader health context. The research purpose was to contribute to the body of indigenous knowledge systems to advocate towards co-existence of primal health care and mental health care.

**Aim:**

Formulate APHC within a mental health care context.

**Objectives:**

To explore philosophical grounding of APHC and describe epistemology of APHC. To analyse and crystallise the exploration to establish understanding within mental health and conceptualise APHC within mental health care to enhance co-existence.

**Methodology:**

Narrative synthesis, concept analysis (qualitative design). Lekgotla was used as a method of data collection and data were analysed using Leedy and Ormrod’s five steps of data analysis.

**Results:**

APHC is a health care system that existed in Africa prior to the introduction of the western health care system. It is based on the African belief system and practices. The practices come from the community, for the community and are authenticated by the community. APHC uses a holistic approach and the family and community are involved in the healing process.

## Introduction

Mental health disorders affect more than 25% of all human beings at some point in their life (WHO [World Health Organization] 2001, cited in Wang et al. 2007). It is estimated that 450 million people worldwide have at least one mental disorder, and 20% of all patients examined at primary health care services are diagnosed with one or more mental health disorders (McBain et al. 2012:444). In addition, there is high incidence of mental health disorders (25%–64%) in individuals treated for physical illnesses in the primary health care settings in the United States (Kneisl 2013a:6). Subsequently, the incidence of mental illness in South Africa is similar (Lund, Thomlison & Patel 2016:1; Miya 2016:18).

Adding to the mentioned factors is the fact that neuropsychiatric disorders are a leading cause of disability worldwide and account for 37% of all loss of life (Wang et al. 2007:841). In 2000, mental health accounted for 12% of the global burden of disease (Lund et al. 2007:352). However, only one-third of people suffering from mental disorders receive treatment for mental illness in high-resourced countries and as little as 2% in some low- and middle-income countries (Eaton et al. 2011:1593). Even families with medical insurance find themselves responsible for the mental illness treatment costs as not all insurances cover mental illnesses (WHO 2001:24). Less progress in response to mental, neurological as well as substance misuse disorders is observable, especially in the poorest countries and regardless of the reportedly large treatment gap identified (Eaton et al. 2011:1592).

Consequently, Kneisl (2013a:8 cited in Prince et al. 2007) argues that the burden of mental disorder is underestimated because of the fact that there is little appreciation of the connection between mental illness and other health conditions. Hence, unequal service delivery is provided in primary health care facilities, with reproductive health care, child health care and physical health being given more attention in comparison with mental health care (Mangula & Pienaar 2013:146). Therefore, it is a common course that this practice in the western health system poses severe challenges for holistic care, including mental health challenges.

## Background

Problems, for example medication stock-outs, staff shortages and long waiting time, are encountered when access to mental health care is needed in primary health care facilities (Kneisl 2013a:6). According to Thom (2004:33) and Ssesbunnya et al. (2010:117), there is a constant loss of experienced professionals, and, within the African continent, there are few trained mental health professionals (Atindanbila & Thompson 2011:457). An average number of health and mental health professionals in all countries was found to be low, and almost half of the countries (90% of African countries and all SouthEast Asian countries) are reported to have less than 1 psychiatrist per 100 000 people (Jacob et al. 2007:1061). The ratio for indigenous practitioner per community is 1:200 as compared to 1:100 000 for western-trained medical doctors and psychiatrists, respectively (Atindanbila & Thompson 2011:459). Psychiatrists are rare in low- and middle-income countries and are expected to assume multiple roles of diagnosing and treating patients and training staff members while managing the facility at the same time (McBain et al. 2012:445).

What is of concern to Shai-Mahoko (1996:31) is that even with such clear evidence of the western health care struggle, indigenous practitioners are still denied recognition and active involvement in the provision of community health care and that their practice is instead ridiculed. Van Rooyen et al. (2015:2) reported that South Africa is experiencing an increasing number of people who make use of both the western health system and indigenous knowledge system (IKS). It is clear that there is a greater number of indigenous health practitioners than western-trained practitioners and that the burden on the primary health care system could be alleviated if primal health care is understood and can formally co-exist with primary health care. Therefore, this research seeks to conceptualise the concept of African Primal Health Care (APHC) from a foundational, philosophical context.

## Aim and objectives of the research

### Aim

The aim of the research was to formulate the concept, APHC, within a mental health care context. In order to achieve the main aim of this study, the following objectives were developed: exploring the philosophical grounding of APHC; describing the epistemology of APHC; analysing, synthesising and crystallising the above-mentioned exploration in order to establish an understanding within mental health; and conceptualising the concept, APHC, within a mental health care context to enhance co-existence.

### Research design

According to Brink, Van der Walt and Van Rensburg (2014:113), qualitative research is a type of research design that is used to explore, describe and promote an in-depth understanding of human experience. As a research method, the researcher used narrative review and narrative synthesis to conceptualise the concept, primal health care, in an African context. The study utilised qualitative narrative synthesis as the design enables the readers to have insight on how explanations and empirical findings have linked and changed one another through time (Mays, Pope & Popay 2005:12). Narrative synthesis is used as an aim to develop theoretical models; to identify, explain and give insights on complicated or controversial issues; to share information that can assist in improving practitioners’ best practice; and to bring new insight of emerging issues (Denyer & Tranfield 2006:219). Primal health care is a controversial issue because it competes with primary health care and community health care.

Although narrative synthesis is often viewed as open to interpretative bias and less rigorous, it offers a more holistic and inclusive way of viewing studies (Waring et al. 2010:27). Furthermore, it accommodates qualitative description, synthesis and interpretation rather than testing existing theories, which leads to the identification of themes (Waring et al. 2010:27). Moreover, Mays et al. (2005:11) assert that narrative synthesis interprets, explains and summarises evidence of a phenomenon. In addition, narrative synthesis deals with the findings and interpretation from published studies and other sources in their own terms without attempting to transform them into a common metric for analytic purpose (Mays et al. 2005:12).

Mays et al. (2005:12) elucidate that there is a distinct difference between narrative review and narrative synthesis and explain that, unlike narrative review, narrative synthesis goes beyond just summarising the study findings and attempts to bring new insights or knowledge while also being more systematic and transparent. Narrative synthesis refers to the research design process undertaken by the researcher to synthesise evidence taken from multiple studies or contexts (Denyer & Tranfield 2006:219; Harrison et al. 2012:337; Mays et al. 2005:12).

**FIGURE 1:**
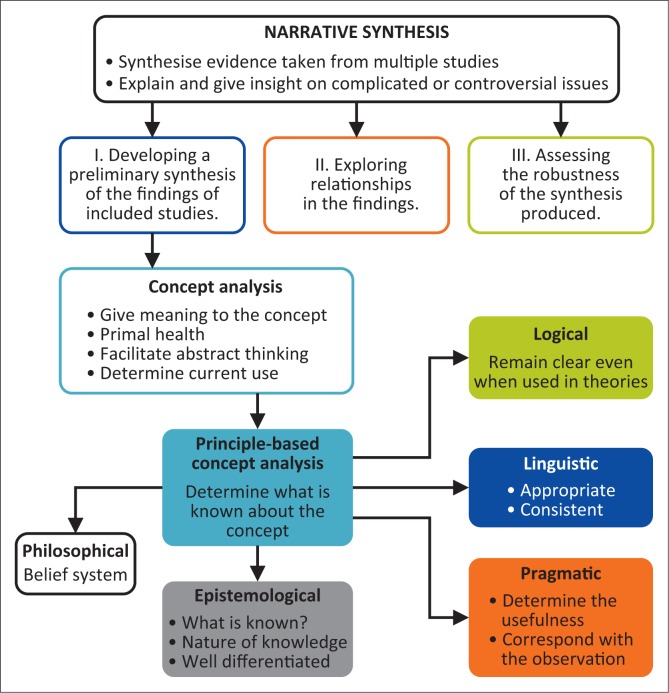
Conceptualisation: African primal mental health care.

Walker et al. (2010:744) suggest six steps of narrative synthesis, whereas Mays et al. (2005:12) reduce the six steps to only three steps. The narrative synthesis process in this study was explored in three steps that are common in both studies: The first step consisted of developing a preliminary synthesis using a concept analysis method; the second step involved exploring relationships of the findings; and the third and final step assessed the robustness of the synthesis produced. Then, data were crystallised leading to conceptualisation (see Figure 1).

### Setting, population and sampling

The population of this study comprised the Seboka team members. Seboka team is a community based indigenous research project under the IKS project (project number: NRF IKP 20701130000018563). The team is a multi-country collaboration research team consisting of various African indigenous researchers: beginner researchers (honours and master’s students), novice researchers (PhD candidates) as well as senior research collaborators across various southern African countries (South Africa, Malawi, Zambia, Zimbabwe and Tanzania). This team holds at least one capacity-building workshop a year, and the retrospective data of the workshops are available to all team members. This research used the retrospective data collected, respectively, in Lesotho, Tanzania and Kimberley in South Africa. The discussion was based on the concept, primal health care, and the colloquium was facilitated in the form of a *Lekgotla* (for more detail, see the section ‘Data collection and data analysis’).

The team consisted of researchers who focused on contemporary issues regarding IKSs, which is in line with the phenomena of interest. There is a belief that the larger the sample size, the better the results. This holds true to some extent in quantitative study, but is not applicable to qualitative studies because with qualitative designs, once a specific size is reached, the researcher will not improve the results significantly by increasing the sample size (Brink et al. 2014:135).

### Non-probability sampling approach

Non-probability sampling is a type of sampling approach that may or may not represent the population accurately (Brink et al. 2014:131; Burns & Grove 2009:353). In addition, a non-probability, purposive sampling approach is an approach that is used to select participants who are knowledgeable about the phenomenon and usually more convenient for the researcher if he or she is unable to locate the entire population because of limited access (Brink et al. 2014:131). The Seboka team consists of researchers around southern Africa, and during the colloquia, not all members are able to attend because of administration challenges. Therefore, any willing team member who attended any of the three capacity-building workshops took part in the research.

The researcher carefully selected data sets of research colloquia that are more relevant to the phenomena of the study within the Seboka (Brink et al. 2014:132). The Seboka team members are regarded as the most knowledgeable subgroup about the African primal health concept, and the team discussed the concept of primal health care during their capacity-building workshop in Lesotho and Tanzania (Seboka team 2013; Taaka et al. 2013:128). The term ‘primal’ was coined during a colloquium in Lesotho by Dr Mbulawa and the Seboka team members. Data saturation was reached by the third colloquium, which was held in the Northern Cape, South Africa (Brink et al. 2014:135). Table 1 represents the sampling for the three colloquia.

**TABLE 1:** Primal health participants.

Variables	Lesotho	Tanzania	South Africa
Number of participants	10	15	25
Age of participants	30–40 years: 4 41–50 years: 5 60–70 years: 1	20–30 years: 3 31–40 years: 6 41–50 years: 5 60–70 years: 1	18–20 years: 1 20–30 years: 5 31–40 years: 5 41–50 years: 10 51–60 years: 3 61–70 years: 1
Gender of participants	6 males 4 females	8 males 7 females	15 males 10 females
Race of participants	9 Africans 1 white	10 Africans 1 mixed race 4 whites	16 Africans 4 mixed race 5 whites

### Data collection and data analysis

Conducting and documenting direct observations of the events and actions as they naturally occur is one of the approaches recommended by several researchers (Yin 2013:322 cited in Erickson 2012:688; Maxwell 2004; 2012; Miles & Huberman 1994:132).

The researcher used *Lekgotla* as a technique to collect data (Pienaar 2015:59). Pienaar (2015:59–64) defines *Lekgotla* as a form of qualitative data gathering relevant for research conducted with the community. The latter evolved as an attempt to give the community back their dignity, which can be lost through research, by giving them a voice to tell their own story. During *Lekgotla*, one person, usually the chief, chairs the discussion, and every member in the discussion gives their opinion and expertise (Motshegare 2013). Pienaar (2015:59) further alluded that *Lekgotla* as a data collection method adheres to the most important principle of allowing the research to occur in the participants’ natural setting and, as a result, allows more information to be obtained from participants. For the purpose of this study, *Lekgotla* was applied differently as the colloquia only included researchers who are more knowledgeable with regard to the IKS and primal health care concept and was led by two senior research collaborators instead of the chief. It is worth noting that even though the setting of the *Lekgotla* was different in this study, the principles of *Lekgotla* were adhered to. The researcher was part of the gathering as a participative observer. The researcher used audio and video recordings during *Lekgotla* to collect data, and field notes were also taken during the process, included as data and analysed (Bloor & Wood 2006:28; Leedy & Ormrod 2010:137).

The researcher transcribed the data, including recordings (video and audio) and field notes. Then, the researcher initiated data analysis by looking for themes which fit into the principle-based concept analysis within the raw data and then compared the themes found (Bloor & Wood 2006:28). Leedy and Ormrod (2010:138) cited Creswell (1998) and Stake (1995) who recommended five steps for data analysis, which were applied in this research:

**Step 1**: The researcher organises the facts found about the case in a logical manner.
**Step 2**: Findings of categories from the data are arranged into themes.
**Step 3**: Data are analysed for specific meanings that can relate to the case.
**Step 4**: Data that are interpreted are examined for underlying themes and patterns.
**Step 5**: Conclusions about the data are made in a form of narrative storytelling.

### Ethical considerations

The research proposal was submitted to the Research Ethics Committee School of Nursing Science at North-West University for approval prior to the commencement of the study, permit number: NWU-00052-16-S1. As a Seboka team member, permission was also requested from the Seboka senior research collaborators to use the data set collected during the Makgotla (colloquia) from the Lesotho and Tanzania workshops in their discourse as the researcher was a participant observer. Moreover, a Seboka code of conduct was signed by the researcher and each member of the team.

Institutional permission was obtained to conduct the study. The researcher followed the five general ethical principles stipulated by the American Psychological Association (APA) (2010:3–4) and applied them in the study as elaborated in Table 2.

**TABLE 2:** Ethical considerations.

General principle	Application
Beneficence and non-maleficence	A full explanation of the study was given to the participants, including the risk and benefit ratio. Participants were also informed that participation is voluntary and that they may withdraw from the study without any prejudice to them.
Fidelity and responsibility	The researcher is part of the Seboka team as a beginner researcher and thus had access to the information discussed during the *Makgotla* (singular: *lekgotla*). As part of the project regulation, all members sign a code of conduct upon joining the team. The code of conduct states the focus of the research project (community based or indigenous) as well as the role of each team member. The researcher ensured that the participants of the colloquia comprehended this information prior to participation (Botma et al. 2010:11–12).
Integrity	To ensure integrity, the researcher strived to promote accuracy, honesty as well as trustworthiness: Data were documented accurately and completely. Correctness of the transcripts was checked by using the coding system; co-coding was done by a person with a master’s in psychiatric nursing. Triangulation of different sources of data and examining the evidence by checking the extent to which conclusions, based on quantitative sources, are supported by a qualitative perspective and vice versa (this was achieved by using different data sources of information).
Justice	The term ‘primal’, meaning original, was coined during one of the Seboka colloquia by Dr Mbulawa and the Seboka team members. Therefore, participants were selected because they were considered to be most knowledgeable about the concept of APHC. Data were recorded and safely stored in a steel cabinet at the School of Nursing; a soft copy was also safely stored in an institutional password and virus protected computer to which only the researcher had access.
Respect for people’s rights and dignity	The researcher also ensured that the rules of the colloquia were obeyed by signing the Seboka code of conduct, and the participants’ rights were protected, including the right to self-determination, the right to privacy, the right to anonymity and confidentiality, the right to fair treatment and the right to be protected from discomfort and harm (Botma et al. 2010:11–12; Brink et al. 2014:32–40; Burns & Grove 2009:195). Only the name of the team was used and no individual names are mentioned in the publishing of the research work.

## Findings

From the data analysed and crystallised, eight attributes of primal health care evolved. An overview of these attributes is provided in Table 3.

**TABLE 3:** Findings of the study.

Primal health care attributes	Application of attributes
Foundation in the African philosophy of Ubuntu and the belief system	With primal health care everyone is included in the healing process because an injury to one is an injury to all and with Ubuntu: ‘I am because you are and you are because I am’.
Uses holistic approach, not compartmentalisation	The healing process is not separated into mental or physical health but rather intertwined in a holistic approach. The healing goes to the extent of not only including the ill individual but also involving the family and, at times, the whole community.
Primordial system	Primal health care is the health care that originated in Africa and was practised in Africa prior to the colonisation era and introduction of the modern health care system.
Mobilises support from within the family and community	The family and community plays an important role in the healing process as the ill individual is nursed at home and not institutionalised.
Practised in the community	Indigenous African communities believe in the bond between the living and the ancestors and that indigenous practitioners have the calling or gift to connect the two. The connection process is done by the chosen community indigenous practitioners in the community for the community.
Validated and confirmed by the community	Before any use of a medicinal plan, the selected community members undergo an experimental journey where a sick animal eating that particular plant is observed and monitored for the healing process.
Based on an acceptable process of learning, approved and accepted by community	The indigenous student known as *mathwasana* undergoes intense training before he or she can start to facilitate any healing process. Not everyone can become an indigenous practitioner as it is a calling.
Uses natural resources available in the community	African indigenous community make use of natural resources such as medicinal plants to facilitate healing.
Supersedes primary health care and is cost effective	Consultation of the indigenous practitioner is more affordable (payment is usually in kind and not in cash) and often the ill person only pays after he or she has recovered.

## Discussion

Because the study followed a narrative synthesis, the findings are discussed according to the three steps as stipulated in the methodology section. The first step consisted of the researcher developing a preliminary synthesis of the findings of included studies through concept analysis.

### Concept analysis

Within concept analysis, the researcher gave meaning to the concept ‘primal health’, facilitated abstract thinking and determined current use. The latter was done by first giving a theoretical definition of primal health care elaborating on the principle-based concept analysis using the five attributes: philosophical, epistemological, pragmatic, linguistic and logical principle-based concept analysis.

### Theoretical definition of primal health care

Western health care refers to the organised medical care that is given to an individual or community (Concise Oxford English Dictionary 2008:658). Taaka et al. (2013:128) and Bailliere (2009:69) further elaborated that with health care there is continuous provision of welfare and protection, which leads to promoting well-being and preventing any disorders, including mental disorders.

During the colloquia, the team concluded that:

African Indigenous health means a healing process practiced within a specific community by means of divination. The healing process is different from community to community and thus the use of medicinal plants is also different (that is, the same plant can be used for different healing purposes by different communities).

Perusing these definitions, health for the researcher means a holistic healing approach within a community which focuses not only on the individual but also on the family and community at large. Within the healing process, divination and the use of medicinal plants are included, and it must be noted that the healing process is highly spiritual and sacred.

Derived from the Latin word *primalis* or *primus*, ‘primal’ means *‘*first’, and it relates to an *early stage in revolutionary development* (Concise Oxford English Dictionary 2008:1139). Primal, which also means primordial, refers to ‘original’ (Freshwater & Maslin-Prothero 2005:479). Prior to the evolution of modern health medicine, African indigenous individuals used indigenous health care services; this included consultation or visits to the various indigenous practitioners (Gumede 1990:45). This health care according to the Seboka team, including the researcher, is known as primal health care. Hence, primal health care was the healing system used by indigenous Africans prior to the introduction of the modern health care system (Taaka et al. 2013:128). These authors further cited *Mosby’s Dictionary of Medicine, Nursing and Health Profession* (2009) and stated that primal is something that comes from the mother. The African community refer to their continent as mother Africa. Therefore, she (mother Africa) takes care of her African children by giving them medicinal plants and animals to heal (*ibid*). As a result, indigenous Africans use the field as their pharmacy to obtain medicinal plants to facilitate the healing process. Conversely, healing within primal health care is holistic and intertwined and thus involves several processes such as spiritual divination [such as *ditaola* (bones) and taking of medicinal plant remedies]. Within primal health care, healing is unique and locally practised from clan to clan, and the knowledge is transferred from generation to generation through an initiation process called *ukuthwasa* in isiZulu (meaning ‘training to become a traditional healer’).

As stated, thus far, primal health care takes place within an indigenous community and starts with the delivery of health care by fellow community members, the elders and indigenous health care providers.

After the theoretical definition was explored, the researcher went further and analysed primal health care based on principle-based concept analysis using the five attributes, namely philosophical, epistemological, pragmatic, linguistic and logical principle-based concept analysis.

## Principle-based concept analysis

### Philosophical principle

Primal health care’s philosophical principles were analysed based on the African context as the study is on an IKS. The concepts used to explain the African belief system are the person, his or her family and the community, and because the concept includes health care as an aspect, health, illness and healing are included as well. The person within the African context is a being not only consisting of flesh and blood but also existing as a whole, including his or her mind, body and soul. However, in Africa, no man exists in a vacuum. Therefore, the family members play a crucial role in each other’s lives. However, the family not only includes the nuclear family members but also involves uncles and aunties as well as cousins. The saying *Motho ke motho ka batho babangwe* (‘I am because we are’) is the anchor of African communities, and the community considers themselves as one. When any member of the family or even a community passes on in Africa, it is believed that they cross over to another world where they become the protectors and providers of spiritual guidance to the family and community. It is believed that there is a special bond between the living and the deceased, and dreams are used as a medium of communication between the two worlds.

With the special bond included, Africans make use of indigenous practitioners to facilitate the healing process where practices such as divination are highly practised. Apart from dreams, the throwing and reading of bones (*ditaola*) is also used to spiritually deal with disharmony (illness) and thereafter to facilitate healing.

The above findings concur with that of Phiri and colleagues, as the authors stipulate that:

In Africa, proverbs are used as a form of teaching to help us make interpretations of our everyday existence through dialogue and individuals’ collective wisdom which is transmitted from one generation to the other, providing insight into how people live and behave (Phiri, Mulaudzi & Heyns 2015:1).

It is through proverbs that African men and women shape their lifestyles.

### Epistemological principle

‘If you kill a cat to see how it functions, the first thing you have is a non-working or dead cat’ (Participant 1, male, research collaborator).

The above statement refers to the holistic practice of the indigenous community within the primal health care system. This finding is confirmed by the following direct quotation from a transcript:

‘The west take one thing and commercialise it and do not look at the holistic practice…. Like they are doing to our medicinal plants, they take one active ingredient and leave the rest out that is why their medication has such severe side effects’ (Participant 2, male, novice researcher).

Indigenous philosophers do not separate physical and mental illness, but rather believe that when an individual is ill, the illness affects the person as a whole (Van Dyk 2005:195). African indigenous practitioners commonly locate the cause of psychological distress within the community and thus base the management within the community as well (Mzimkulu & Simbayi 2006:418). Some of the features of mental illness common in African indigenous communities which were reported by indigenous practitioners include bizarre content of speech, running away from home, talking alone or laughing inappropriately (when there is absolutely nothing funny around), undressing or removal of clothing items everywhere and poor personal hygiene (Ovuga, Boardman & Oluka 1999:277). These features are commonly known to indigenous healers and the indigenous communities as *amafufunyane* and *mafonfonyane* in isiXhosa and Setswana, respectively (Mzimkulu & Simbayi 2006:418; Pienaar & Manaka-Mkwanazi 2004:130).

The statement below came up when one of the participants explained that as an indigenous knowledge descendant growing up in a primal health care setting, he was exposed to the practices and could give information on the practices without even practising. Observation was the most important aspect in primal health. The participant said:

‘During our training as indigenous practitioners, we look at a sick animal, we follow the animal, look at the type of plants it eats and look the rate of its recovery. We then take the samples of those plants and experiment with them first on ourselves, then others. Therefore, if there is such a thing as science, then indigenous knowledge is science as there is logic and it is systematic and there are experiments done.’ (Participant 3, male, research collaborator)

To correct the misconception regarding indigenous healing as a healing for uneducated people, Thornton (2009:19) made it known that there is a formal education system that involves teachers known as *gobela* and students known as *mathwasana*. At the end of the formal teaching period, there is a graduation ceremony that certifies *mathwasana* as *sangomas* or indigenous healers (Thornton 2009:18). Indigenous knowledge is acquired through observation and practice where *mathwasana* first observe the *gobela* and thereafter practise with several community members under supervision where the outcomes are ratified and critique is delivered by the *gobela* (Thornton 2009:25). The indigenous health care user remains the decision-maker of his or her healing process and should decide whether he or she is satisfied with the service the student provided and should there be dissatisfaction of any kind, the *gobela* will take over (Thornton 2009:25). Not everyone can become an indigenous practitioner as it requires an ancestral calling and skills training (Taaka et al. 2013:126). Care within primal health care is given at home or in the community and is given in a personalised manner (Valfre 2013:12; 33). Prior to the evolution of modern health medicine, African indigenous individuals made use of indigenous health care services where the use of indigenous medicinal plants was high in practice (Gumede 1990:45). Although most western-trained health care providers assume that crude medicinal plants interfere with modern medicine, most medicinal plants used by indigenous practitioners have nutritional value and do not interfere with any function of modern medicine (Taaka et al. 2013:128). *Lengana*, for example, one of the medicinal plants commonly used by indigenous practitioners, is high in protein and fat and is given to psychotic individuals to replace the energy used during episodes of psychosis (Taaka et al. 2013:127).

Just as is the case with modern medicine, indigenous practitioners make use of measurements when giving medicinal plants (Taaka et al. 2013:126). Instead of using milligrams and millimetres as a measuring guide, some of the measuring methods Africans use include the fingers and the palm of the hand, which they believe are directly proportional to the body mass index (Taaka et al. 2013:126).

### Pragmatic principle

Most countries practise their own form of indigenous healing, which is firmly rooted in their culture and history.

The quote by Participant 3 in the ‘Epistemological principle’ section also serves to explain how the indigenous communities determined the usefulness of medicinal plants in the absence of laboratory *in vivo* and *in vitro* studies.

Indigenous healers use various methods to heal. The following practices are the most commonly used methods: 80% home fortifying and home cleansing, 77% personal cleansing, 68.5% scarification and 60% cultural education, which includes, but is not limited to, taboos and sexual education (Shai-Mahoko 1996:32). Indigenous healers therefore offer a wide gamut of counselling and divination (diagnostic) services (Thornton 2009:17).

The indigenous African society believes in the bond between the living and the ancestors and that indigenous practitioners have the gift and ability to connect the consulting individual and his or her ancestors through the spirit (Degonda & Scheidegger 2009:1; Gumede 1990:10; Marsella & Yamada 2000:17). The importance of the connection between the ancestor and the indigenous health user is based on the belief that ancestral spirits make their presence felt by causing illness in the particular individual or family and also that disease is man-made and uses the spirit as an agency of transmission (Gumede 1990:19 & 41; Pienaar & Manaka-Mkwanazi 2004:130).

In the African indigenous community, consulting an indigenous practitioner works both ways: either the sick but stable individual goes to the indigenous practitioner’s practice room (usually a hut within the family yard) or the indigenous practitioner goes to the consultee because the condition of the consultee is critical, the individual does not have enough money for transportation or because the ancestors instructed the indigenous practitioner to do so (Pienaar & Manaka-Mkwanazi 2004:135; Sorsdahl et al. 2010:286).

Mental health care users do not optimally utilise the western health care system because their first point of consultation is with the indigenous practitioner and they only consider modern health care when the condition does not improve or when it worsens. In addition, they still go back after discharge to consult with their indigenous practitioners as they need to be cleansed for the curse they believe was inflicted on them (Gumede 1990:39).

### Linguistic principle

‘Ubuntu, I am because you are, and you are because I am and because you are, I am and therefore we are… Ubuntu – the bed rock of indigenous knowledge’ (Participant 4, male, novice researcher).

The above quote, shared by a participant, indicates that holism within the indigenous system is not only limited to the community but also to the cosmos at large. The author would further like to emphasise the two common terms within primal health care identified by participants, namely ‘knowledge holders’ and ‘knowledge carriers’: ‘Knowledge holders can be taught the knowledge but knowledge carriers are born with the knowledge and it (knowledge) may or may not manifest itself’ (Participant 5, female, novice research).

‘The clay pot in an indigenous way or in the Sesotho way of doing things normally contains the African beer and in that clay pot, each and every one who is around shares the beer from the same clay pot.… The clay pot symbolises sharing … it is communal solidarity.’ (Participant 6, male, beginner researcher)

The latter is in line with what Mulaudzi and Peu (2013:3) stated in their study, namely that Ubuntu is anchored in the principles of solidarity, consensus, sense of belonging as well as participatory decision-making. They clarified that Ubuntu does not take away the uniqueness of the individual but put emphasis on neighbourliness where there is a shared goal and sharing of resources.

### Logical principle

During consultation with the indigenous practitioner, detailed history taking, physical examination and divination are common ways utilised to make a diagnosis (Atindanbila & Thompson 2011:460; Ovuga et al. 1999:277). The African indigenous healing system focuses mainly on the ‘whom’ instead of the ‘what’ way of healing, and the main question is whether the cause was angry ancestors, evil spirit(s) or bewitchment (Mzimkulu & Simbayi 2006:420).

This discussion marks the end of step 1, which was followed by step 2 of the narrative synthesis during which the researcher explored relationships in findings. Primal health care, as a controversial issue, competes with primary health care and curative hospital-based care. For the purpose of co-existence, the author saw fit to undertake this exploration. The exploration is presented in Table 4, focusing on three aspects, namely identification of the reason for consultation, disease management and practices.

**TABLE 4:** Exploring relationships in the findings.

Healing process	Primal health care Communal-based (family, community and indigenous clan [society])	Primary health care (Western community establishment-based)	Curative hospital-based health care (Western institution-based)
Identification of the reason for consultation	During consultation with the indigenous practitioner, detailed exploration, physical examination as well as divination are the common ways of finding a diagnosis (Atindanbila & Thompson 2011:460; Ovuga et al. 1999:277). In most cases, before an individual even considers to go consult an indigenous practitioner, he or she has already been visited by the ancestors to inform him or her about that individual. By the time that individual decides to consult the indigenous practitioner, the practitioner will already be awaiting him or her. A time frame is given and if that time passes without that person consulting, the indigenous practitioner will then visit the person at her or his home depending on the severity of the condition and the urgency thereof.	Valfre (2001:34) explains that theory about illness is based strictly on scientific findings and thus the management thereof has to be scientifically proven as well tending to discard any management that cannot be scientifically proven. An individual has to have a bacterial, fungal or viral infection to seek consultation and symptoms must be present (tendency to not treat asymptomatic individuals except in exceptional cases).	With western health care there are rules that determine the appropriateness of social and ethical behaviour and if an individual acts beyond what is considered legal behaviour, mental illness is then diagnosed (Kneisl 2009:6; Sadock & Sadock 2003:16). Only two psychiatric diagnoses were recorded by health care professionals, namely schizophrenia and epilepsy, which questions the knowledge and competence of the practitioners (Modiba et al. 2001:192). The system prefers mostly referrals from primary health care with evidence that initial attempt was made to manage the illness prior to referral.
Disease management	Believes that illness is the result of bad spirit, thus consultation of indigenous practitioners is preferred to chase the spirit away and restore wellness through cleansing (Atindanbila & Thompson 2011:458; Gumede 1990:10; Kigozi 2003:27; Marsella & Yamada 2000:17; Ssesbunnya et al. 2010:130). The whole family and, in some instances, the community is given treatment. Instructions are given to the family members to facilitate the healing process especially if the consultee is critically ill. Medicinal plants, hygiene and nutrition become the priorities followed by cleansing when the individual has recovered or is recovering. In some instances, the indigenous practitioner will take the ill individual to his or her home where the indigenous practitioner’s family and students (*mathwasana*) will care for the ill or the indigenous practitioner will move in to the ill person’s home until recovery is obtained.	There are specialities within the health system, for example psychiatry, oncology, medical, surgical, with a focus on the specific main complaint. Illness tends to be reduced to a particular disease, with the emphasis on the pathophysiology and the body mostly given sole attention instead of regarding the individual as a whole (Kneisl 2013b:168). Medication given is for the management of the presenting symptoms.	The hospitals are understaffed and over populated in such a way that available staff on duty can only focus on performing what is considered essential nursing tasks such as giving of medication and as a result neglect psychotherapies and occupational therapies (Janse van Rensburg 2010:385).
Practices	There is a substantial imbalance between urban and rural community health services in Africa and this leads to the health services remaining under developed and the well trained and specialised personnel relocating to the west (Atindanbila & Thompson 2011:457; Kigozi 2003:1 & 2). Decentralisation of mental health care users to the community is given priority; however the community is not approached to be made aware and also the liaison services are lacking (Kigozi 2003:1). Sub-Saharan region seem to not be aware of the seriousness of mental health burden as the epidemiological studies available are limited. Indigenous health care is more affordable and accessible compared to the modern health care system (Atindanbila & Thompson 2011:459; Degonda & Scheidegger 2009:2). The cost within the primal health care system is cheaper and more flexible to accommodate the community; more often than not, the person only pays once full recovery is obtained.	The use of the Band-Aid approach, treating only the presenting complaint (Valfre 2001:12). Make use of the same medicinal plant, synthesise it chemically, then only take the active ingredient and make it in a form of tablet, capsule and so on (Taaka et al. 2013:127).	According to Janse van Rensburg and Jassat (2011:25) supported by Janse van Rensburg (2010:386), 17.3% of mental health care users in Helen Joseph Hospital had exceeded the expected period of admission, that is, 25 days, whereas 8% mental health users were re-admitted several times during a 12-month period. Kruger and Lewis (2011:127) suggest that an increase in re-admission rate to secondary and tertiary psychiatric level might be mostly because of unsuitable community care facilities rather than the mental health user’s condition. ‘Readmissions also imply that inadequate follow-up of these users in community psychiatric services should be addressed’ (Janse van Rensburg 2010:388). Ramlall, Chipps & Mars (2010:668) mentioned that health care professionals could not comply with the mental health care Act as they had no sufficient beds and the staff was having challenges managing disruptive mental health care users. Furthermore, 69.4% did not have sufficient medical and nursing staff to provide the standard mental health required. This forced the hospital to rapidly and prematurely discharge mental health users who were not really due for discharge and this led to relapse (Van Heerden et al. 2008:4). According to Thom (2004:33), there is constant loss of experienced professionals, and there are few mental health professionals trained in the continent (Atindanbila & Thompson 2011:457). Another constraint is that most of the time the facilities cannot meet the needs of the mentally ill individual as personnel available are not trained or well equipped to work with mentally ill individuals and, more often, professionals who are trained and equipped are unwilling to work with mentally ill individuals (Van Heerden et al. 2008:4).

*Source*: Authors’ own work

### Robustness of the primal health care

Regarding step 3 of the narrative synthesis, the author identifies the strengths of primal health care towards co-existence. Primal health care practices come from the community, are to be used within the community and have to be authenticated by the community. In addition, primal health care uses a holistic, communal approach of healing in which the family and community at large are involved in the healing process. Furthermore, the healing process not only focuses on a specific part of the body but embraces the person as a whole (mind, body, spirit and cosmos). Not everyone can become an indigenous practitioner as it requires an ancestral calling and skills training (Taaka et al. 2013:126). An indigenous scholar must undergo an extensive process of learning to heal prior to joining the practitioner fraternity. Natural resources such as medicinal plants, which are available within the community, are used to facilitate healing. The services provided during primal health care consultation are cost effective and readily available (such as medicinal plants and the indigenous practitioner’s home) and are more often than not the first contact before modern health care services are considered as well as the last one to complete the healing process by means of a cleansing ceremony.

After the third step of narrative synthesis, the author crystallised the data. When multiple methods of data collection and analysis are used to validate the results, the process is called crystallisation; it gives a complex and more deepened understanding of the phenomenon (Maree & Van der Westhuizen 2007:40). Nieuwenhuis (2007:81) indicates that crystallisation aims at allowing the researcher to shift from viewing things in a fixed and rigid manner. Maree and Van der Westhuizen (2007:40) emphasise that it is of utmost importance for the researcher to attend to voices which differ from his or her own in order to learn more about multiple constructed realities. The different insights gained give different perspectives that show the uniqueness of the realities and identify participants (Nieuwenhuis 2007:81). According to Maree and Van der Westhuizen (2007:39), in order to interpret validity and establish data trustworthiness, the researcher needs to consider triangulation which was achieved by checking the extent to which conclusions based on quantitative sources are supported by a qualitative perspective and vice versa. This was achieved by using different data sources of information (Creswell 2014:191). Crystallisation of data in this research occurred when the researcher used the data collected from the three colloquia and the existing literature to gain more insight from those sources; a more in-depth understanding of the concept, APHC, was established. Crystallisation of data led to the conceptualisation process, and as a result, the attributes stipulated in Table 3 evolved.

## Conceptualisation

Botma et al. (2010:57) cited in Babbie (2008) define conceptualisation as the mental process where concepts which are viewed as complicated and vague are made to be more precise and detailed. This mental process aims at grasping the specific meaning of the phenomena of interest, which in this study is primal health care (Botma et al. 2010:57).

### African primal health care in mental health care

Lund et al. (2007:352) highlighted that mental health accounted for 12% of the global burden of disease in 2000. Adding to this, Wang et al. (2007:841) revealed that the leading cause of disability worldwide is neuropsychiatric disorders that account for 37% of all loss of healthy life. However, it is still a concern to Eaton et al. (2011:1592) who postulate that less progress has been observed, especially in poorer countries, in response to mental, neurological as well as substance misuse disorders regardless of the large treatment gap identified. Only one-third of people suffering from a mental disorder are treated in high-resourced countries and as little as 2% in some low-income as well as middle-income countries are treated (Eaton et al. 2011:1593). Within primal mental health care, health care is practised within the community for the community, and it is founded within that community’s indigenous practices using available and natural resources such as medicinal plants. The healing facilitated within primal mental health is approved and accepted first by the community and will not only focus on the psychological aspects but also holistically focus on the body, mind, spirit and the surroundings (family, community and ancestors). The family is involved in the healing process and can actively participate throughout the healing process.

According to Saxena et al. (2003) cited in Ramlall et al. (2010:667), 32% countries worldwide did not have a specific mental health budget, whereas 36% only used 1% of the allocated budget on mental health. In KwaZulu-Natal, it was reported that only 0.03% was spent on mental health. The issue of budget is a challenge in all levels of health and does not only affect primary health care services. Hospitals are also affected by this and the latter leads to an unsuccessful integration of mental health and primary health. With primal mental health care, the healing process facilitated and maintained is cost effective as the resources (such as medicinal plants) used in healing are natural and easily obtainable within the community. Psychotherapy is provided by elders within the family with no limit to the number of sessions provided (as in the case of 6–8 sessions prerequisite for modern psychotherapy), and the sessions are also not limited in terms of time but depend on the need assessed for psychotherapy. Even with limited time and a limited number of contact sessions, time is still needed to build rapport before effective psychotherapy can prevail and progress can be made. However, with primal mental health care, rapport is readily available as psychotherapy is not done by a stranger.

As conceptualised in the study:

African Primal mental health care is in brief an African original communal-based health care system that is anchored within the African indigenous belief system and Ubuntu, where an injury to one becomes an injury to the entire community. Within mental health care, the healing process facilitated and maintained is holistic (not separated into mental or physical health but rather intertwined), communal and cost-effective as the resources (human and other health) used in healing are natural and easily obtainable within the community.

## Limitations of the study

As a result of being enrolled for a master’s degree, limited finances and time constraints, the researcher was unable to conduct a multi-context study on concept formulation. A multi-context study has the potential to strengthen the health seeking behaviour, thus leading to health system policy refinement. The former will be bridged by further research. The study was limited to Seboka research team members and three colloquia.

## Recommendations

### Recommendations for health practice

The researcher recommends that the western mental health care system and African primal health care co-exist and should be given equal recognition. Co-existence of mental health and primal health will assist in reducing the number of untreated mental health cases and hospital readmissions. Kneisl (2013a:8 cited in Prince et al. 2007) contends that the burden of mental disorder is underestimated and that this might be because of the fact that there is little appreciation of the connection between mental illness and other health conditions. Nevertheless, the latter will not be a challenge within primal health care as healing is facilitated holistically and not in speciality (compartment). Mental health care users are overlooked because their first point of consultation is with the indigenous practitioner and they only consider the modern health care when their condition does not improve or when it worsens. In addition, they still go back after discharge to consult with their indigenous practitioners as they need to be cleansed of the curse they believe was inflicted on them (Gumede 1990:39).

### Recommendations for teaching and learning

According to Antwi and Ziyati (1993:1), ‘multi-culturalism is a way of creating an open atmosphere for the learning of different viewpoints and the acceptance of these kinds of differences’. Because the nurses are trained to work in an African indigenous community, the researcher recommends that a curriculum should be developed to recognise African primal health system on the same level as the western health care system.

The following can also be done to facilitate co-existence:

- Information sessions can be made to illuminate the concept ‘Primal Health Care in Mental Health’.
- Portfolios, seminars, capacity-building workshops and videos on APHC can be arranged to emphasise co-existence.
- Strategies such as group discussions, role playing and case studies can be developed to strengthen co-existence.
- Students need to be made aware of APHC and its attributes as well as how to merge it with the current modern health care system.

### Recommendations for further research

Further multi-context studies on concept formulation are recommended by the researcher. The research was also limited to mental health care. Therefore, the researcher recommends research into the diverse health care systems.

## Conclusion

The researcher conceptualised African primal mental health care in this research with the aim to advocate for the co-existence of primal health care and other western health care systems in Africa, with specific reference to mental health care. It is noted that African primal mental health care is a communal-based health care; therefore, it is anchored within the Ubuntu philosophy. Communal is deeper than just the community, society or the public as it focuses on a mutual responsibility and collective approach in delivering health care. Therefore, the community is mutually responsible in the healing process, and holistic health care is provided in a family and communal context to the health care user (Paul & Pienaar 2013:40).
